# Unraveling Passivation Mechanism of Imidazolium-Based Ionic Liquids on Inorganic Perovskite to Achieve Near-Record-Efficiency CsPbI_2_Br Solar Cells

**DOI:** 10.1007/s40820-021-00763-8

**Published:** 2021-12-02

**Authors:** Jie Xu, Jian Cui, Shaomin Yang, Yu Han, Xi Guo, Yuhang Che, Dongfang Xu, Chenyang Duan, Wenjing Zhao, Kunpeng Guo, Wanli Ma, Baomin Xu, Jianxi Yao, Zhike Liu, Shengzhong Liu

**Affiliations:** 1 Key Laboratory of Applied Surface and Colloid Chemistry, Ministry of Education, Shaanxi Key Laboratory for Advanced Energy Devices; Shaanxi Engineering Lab for Advanced Energy Technology, School of Materials Science and Engineering, Shaanxi Normal University, Xi’an, 710119 People’s Republic of China; 2 Dalian Institute of Chemical Physics, iChEM, University of Chinese Academy of Sciences, Dalian National Laboratory for Clean Energy, Chinese Academy of Sciences, Dalian, 116023 People’s Republic of China; 3 Ministry of Education Key Laboratory of Interface Science and Engineering in Advanced Materials, Research Center of Advanced Materials Science and Technology, Taiyuan University of Technology, Taiyuan, 030024 People’s Republic of China; 4 Institute of Functional Nano & Soft Materials, Jiangsu Key Laboratory for Carbon-Based Functional Materials & Devices, Joint International Research Laboratory of Carbon-Based Functional Materials and Devices, Soochow University, Suzhou, 215123 People’s Republic of China; 5 Department of Materials Science and Engineering and Shenzhen Engineering Research and Development Center for Flexible Solar Cells, Southern University of Science and Technology, Shenzhen, 518055 People’s Republic of China; 6 State Key Laboratory of Alternate Electrical Power System With Renewable Energy Sources, North China Electric Power University, Beijing, 102206 People’s Republic of China

**Keywords:** Ionic liquids, Inorganic perovskite, Imidazolium, Passivation, High efficiency

## Abstract

The application of ionic liquids in perovskite has attracted wide-spread attention for its astounding performance improvement of perovskite solar cells (PSCs). However, the detailed mechanisms behind the improvement remain mysterious. Herein, a series of imidazolium-based ionic liquids (IILs) with different cations and anions is systematically investigated to elucidate the passivation mechanism of IILs on inorganic perovskites. It is found that IILs display the following advantages: (1) They form ionic bonds with Cs^+^ and Pb^2+^ cations on the surface and at the grain boundaries of perovskite films, which could effectively heal/reduce the Cs^+^/I^−^ vacancies and Pb-related defects; (2) They serve as a bridge between the perovskite and the hole-transport-layer for effective charge extraction and transfer; and (3) They increase the hydrophobicity of the perovskite surface to further improve the stability of the CsPbI_2_Br PSCs. The combination of the above effects results in suppressed non-radiative recombination loss in CsPbI_2_Br PSCs and an impressive power conversion efficiency of 17.02%. Additionally, the CsPbI_2_Br PSCs with IILs surface modification exhibited improved ambient and light illumination stability. Our results provide guidance for an in-depth understanding of the passivation mechanism of IILs in inorganic perovskites.

**Supplementary Information:**

The online version contains supplementary material available at 10.1007/s40820-021-00763-8.

## Highlights

- A series of 10 imidazolium-based ionic liquids (IILs) with different cations and anions have applied to unravel the passivation mechanism of the IILs on CsPbI_2_Br inorganic perovskites.
- It is found that anions of IILs play a more important role in passivation of lead- and cesium-related defects in inorganic perovskite compared with imidazole cations because they can form strong ionic interactions (Pb-F, Cs-F).
- A high-power conversion efficiency of 17.02% is obtained, which is among the highest values of CsPbI_2_Br-based perovskite solar cells.

**Supplementary Information:**

The online version contains supplementary material available at 10.1007/s40820-021-00763-8.

## Introduction

Cesium-based all-inorganic halide perovskites (CsPbX_3_, X = I, Br, Cl, or their mixtures) have recently attracted great attention because of their excellent light stability, heat stability and optoelectronic properties [1–3]. Although great progress has been made in inorganic perovskite solar cells (PSCs) in past two years [1, 4], two core issues limit their future commercialization. On one hand, due to the solution fabrication process and ionic nature of inorganic perovskite, a large number of defects are inevitably formed at the surface and grain boundaries (GBs) of polycrystalline inorganic perovskite film, which serve as non-radiative recombination centers and cause open-circuit voltage (*V*_oc_) and power conversion efficiency (PCE) loss for inorganic PSCs [5]. For example, Huang et al. have demonstrated that the defect density at the interface between the perovskite and hole–transport-layer (HTL) is two orders of magnitude higher than that in the perovskite bulk [6]. These interfacial defects bring in deep electronic states as non-radiative recombination centers, thus restricting the photovoltaic parameters, especially the *V*_oc_ [7–9]. In addition to the efficiency, the inferior phase stability of inorganic perovskite compared to organic–inorganic hybrid perovskites is another serious issue. Researchers also found that defects and water are often involved in the phase transition process of inorganic perovskite film, leading to the instability issue of inorganic PSCs [1, 4]. Therefore, the effective passivation of surface defects and blocking water penetration are especially paramount for simultaneously enhancing the efficiency and prolonging the lifetime of inorganic PSCs.

Surface passivation, for eliminating defects (lattice vacancies, undercoordinated ions and interstitial species) and improving charge transport/hydrophobicity, is one of the most prominent strategies to promote the efficiency and stability of PSCs [10]. Recently, common surface passivators including Lewis bases/acids, organic halide salts, polymers, fullerenes, and their derivatives have been used to passivate the defects of perovskite film [11–15]. Among them, most Lewis bases/acids, organic halide salts and polymers possess insulating properties and could hinder efficient carrier extraction at the interface owing to their poor conductivity, and negatively affect the reproducibility of PSCs due to a distribution in molecular weights [16]. The fullerenes and their derivatives such as PCBM require many steps for synthesis, are expensive, and exhibit low yields [17, 18]. Therefore, the exploration of new passivation materials has become the main topic in research for achieving stable and efficient inorganic PSCs [19].

As a class of molten salts, ionic liquids (ILs) have recently become hot candidates to passivate/modify perovskites and/or charge transport layers for realizing stable and efficient PSCs due to their diverse structures and unique properties, such as wide liquid temperature range, low toxicity, non-volatility, strong conductivity and good stability [20, 21]. Imidazolium ILs (IILs) have unique physicochemical properties, such as versatile functional ligands, large electrochemical window, high thermal stability, and nonhazardous nature, and they have been widely used to passivate the defects both in the interior and at surface grain boundaries of perovskites via additive-assisted techniques or surface-antisolvent methods [22–24]. Bai et al. reported the use of a small amount of the BMIMBF_4_ additive in the perovskite precursor solution [25], where BMIM^+^ in the BMIMBF_4_ can accumulate at the top surface of the perovskite film. The formed BMIMBF_4_ layer could modify the surface dipole and improve the energetic alignment at the top interface of the perovskite film, resulting in reduced voltage loss and improved charge extraction at the interface. In addition, IILs surface passivation of the charge transport layer and perovskite layer has recently emerged as an efficacious strategy to suppress interface defects and reduce interface energy loss in hybrid perovskites [26–28]. For example, Noel et al. recently deposited BMIMBF_4_ on a SnO_2_ electron transport layer (ETL), and it was found that BMIMBF_4_ can simultaneously reduce the work functions of SnO_2_ and perovskite film, thus reducing charge recombination loss and improving the charge extraction and transport at the SnO_2_/perovskite interface [29]. Compared with bottom-surface modification of the ETL, an IIL-based upper-perovskite modification can passivate the bulk defects due to the permeation of IILs into the perovskite layer. Zhang et al. introduced an IIL (1-butyl-2,3-dimethylimidazolium chloride: BMMIMCl) for surface modification of a CsPbBr_3_ perovskite film[30]. It was found that BMMIMCl can passivate the defects (unsaturated Pb^2+^ and Cs^+^) on the surface of the CsPbBr_3_ perovskite film to obtain a PCE of 9.92% in an HTL-free CsPbBr_3_ PSC. Nonetheless, IILs have an electron-rich nitrogen atom, an alkyl side chain and an anion, the crucial interactions between IILs and perovskite precursors are still unclear, and the mechanism of IILs passivation of the inorganic perovskite layer has been rarely investigated. Therefore, further studies are needed to better understand the interaction between the IILs and perovskite and to provide a principle for rational design of the IIL molecules.

Herein, we report a series of IILs as surface passivators for efficient and stable inorganic CsPbI_2_Br PSCs. These IILs possess imidazolium cations with different side chains and different anions. First-principle calculations reveal that anions of the IILs play a more important role in passivation of lead- and cesium-related defects in inorganic perovskite compared with imidazole cations. The anions cause simultaneous ionic bonding of the IILs with Cs^+^ and Pb^2+^ cations on the surface and at the grain boundaries (GBs) of perovskite films, which could effectively heal/reduce Cs^+^/I^−^ vacancies and Pb-related defects. Meanwhile, the IILs could improve the energy-level alignment between the perovskite and Spiro-OMeTAD for promoting hole extraction and reducing electron–hole recombination at the perovskite/Spiro-OMeTAD interface, eventually leading to an increase of *V*_oc_ of 50 mV for the photovoltaic device. Profiting from superior IILs passivation, the efficiency of CsPbI_2_Br PSCs has been elevated from 15.62 to 17.02% with a high *V*_OC_ of 1.33 V. Furthermore, the exposed hydrophobic alkyl component protects the perovskite against detrimental environmental factors. The unencapsulated device modified with BMMIMBF_4_ presents outstanding long-term stability when stored in ambient air at 25 °C with a relative humidity (RH) of 25% or under continuous illumination for 100 h. Our work provides a complete set of characterization methods to elucidate the passivation mechanism of IILs, which provides guidelines for the design of new ionic liquids to improve the performance of inorganic PSCs.

## Experimental Section

### Materials

Cesium iodide (CsI, 99.99%), lead bromide (PbBr_2_, 99.99%), lead iodide (PbI_2_, 99.99%), and lead acetate (PbAc_2_, 99.5%) were purchased from Xi’an Polymer Light Technology Corp. PbI_2_(DMSO), and PbBr_2_(DMSO) were prepared by the antisolvent method. The ionic liquids (ILs) 1-butyl-2,3-dimethylimidazolium tetrafluoroborate (BMMIMBF_4_, 99%), 1-butyl-3-methylimidazolium tetrafluoroborate (BMIMBF_4_, 99%), 1-propyl-3-methylimidazolium tetrafluoroborate (PMIMBF_4_, 99%), 1-hexyl-3-methylimidazolium tetrafluoroborate (HMIMBF_4_, C_10_H_19_BF_4_N_2_, 99%), and 1-hexyl-2,3-dimethylimidazolium tetrafluoroborate (HMMIMBF_4_, 99%) were purchased from Shanghai Chengjie Chemical Co., Ltd. N,N-dimethylformamide (DMF) and dimethylsulfoxide (DMSO) were purchased from Shanghai Aladdin Biochemical Technology Co., Ltd.

### Device Fabrication

The fluorine-doped tin oxide (FTO) glass substrates were sequentially cleaned with ethanol, acetone, and isopropanol in an ultrasonic bath for 30 min and then dried with N_2_. The cleaned FTO glass substrates were then treated with UV-Ozone for 10 min prior to the deposition of TiO_2_. The TiO_2_ layer was deposited by immersing FTO glass substrates in 200 mL aqueous solution with 4.5 mL titanium tetrachloride for 60 min at 70 °C, then rinsed with distilled water and annealed at 200 °C for 30 min. The CsPbI_2_Br precursor solution was prepared by dissolving PbBr_2_(DMSO), PbI_2_(DMSO), CsI, and PbAc_2_ (molar ratio = 1:1:2:0.023) in DMF and DMSO (17:3 v/v). The resulting perovskite precursor solution was spin-coated at 1000 rpm for 10 s, followed by 4000 rpm for 40 s. Afterward, the film was annealed at 35 °C for 6 min, 120 °C for 10 min, and 180 °C for 4 min to obtain the perovskite layer. The IILs were dissolved in isopropanol with different concentrations (0.01, 0.02, 0.03, and 0.04 wt%) and spin-coated onto the CsPbI_2_Br perovskite film at 5000 rpm for 45 s; then the films were thermally annealed at 100 °C for 10 min to form the TiO_2_/perovskite/IIL structure. The hole-transport layer was prepared by spin-coating Spiro-OMeTAD solution (90 mg mL^−1^) doped with 36 µL t-BP and 22 µL Li-TFSI (520 mg mL^−1^) solution in acetonitrile at 5000 rpm for 30 s onto CsPbI_2_Br films to produce a TiO_2_/perovskite/IIL/Spiro-OMeTAD architecture. Finally, an 80-nm Au electrode was deposited by thermal evaporation through a shadow mask to form a device with an active area of 0.09 cm^2^.

### Density Function Theory (DFT) Calculation

The calculation was performed using VASP code in the framework of the PBE approximation. The van der Waals interaction was treated by the Tkatchenko-Scheffler method. The lattice constant of CsPbI_3_ perovskite was set to 6.16 Å, which was obtained by optimizing a cubic CsPbI_3_ unit cell. The interaction energies of the anions in this study with an I vacancy on the film surface were calculated by comparing the energies of a CsPbI_3_ slab (2 × 2 × 3 unit cells in the supercell) with one I vacancy and that of the slab with the I vacancy saturated with the anions. The dimension of the calculation was set to 12.32 × 12.32 × 50 Å^3^. The k-mesh was set to 10 × 10 × 1. In the calculations, the first layer of CsPbI_3_ perovskite was fully relaxed. The interaction energies of the ions of interest with PbI_2_ were calculated in a volume of 20 × 20 × 20 Å^3^. The k-mesh was set to 1 × 1 × 1. In all the calculations, the cut-off energy was set to 500 eV, and the convergence condition was set to 10^–6^ eV to ensure convergence of the system to a stable state.

### Characterization

X-ray diffraction (XRD) patterns of the perovskite films were acquired on a D/MAX 2400 diffractometer. The absorption spectra of perovskite films were measured using a UV–vis NIR spectrophotometer (PerkinElmer, Lambda 950). Photoluminescence (PL) (excitation at 510 nm) spectra were measured using a FLS980 spectrometer (Edinburgh Instruments Ltd), and TRPL spectra were measured with a PicoQuant FluoQuant 300. The scanning electron microscopy (SEM) images of perovskite films were obtained by field-emission scanning electron microscopy (HITACHI, SU-8020). X-ray photoelectron spectroscopy (XPS) and ultraviolet photoelectron spectroscopy (UPS) of the perovskite films were carried out using a photoelectron spectrometer (ESCALAB250Xi, Thermo Fisher Scientific). Fourier-transform infrared spectroscopy (FTIR) were performed with a Bruker Vertex 70. Nuclear magnetic resonance spectroscopy (NMR) was performed using a JNM-ECZ400S/L1 with a frequency of 400 MHz, and deuterated DMSO was used as the solvent to dissolve BMMIMBF_4_ and PbI_2_ with BMMIMBF_4_. The J-V curves of the inorganic PSCs were measured using a Keithley 2400 SourceMeter under AM1.5G illumination at 100 mW cm^−2^. The external quantum efficiencies (EQEs) of the PSCs were recorded using a QTest Station 2000ADI system (Crowntech Inc.). EIS analysis was performed on a Zahner Electrochemical Workstation. Water contact angles were measured using a DataPhysics OCA 20.

## Results and Discussion

**Fig. 1:**
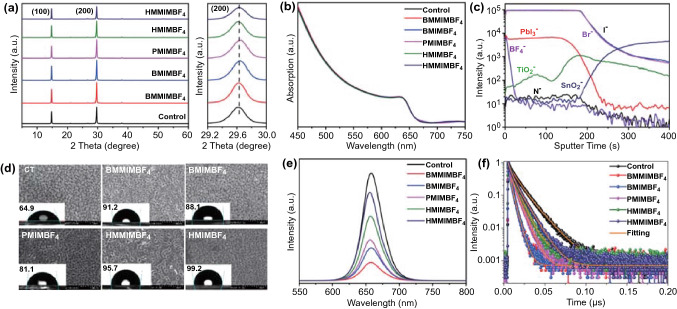
**a** XRD patterns and (200) diffraction peaks and **b** UV–vis absorption spectra of perovskite films with different cation-based IILs treatments. **c** ToF–SIMS depth profile of the CsPbI_2_Br/BMMIMBF_4_ perovskite film. **d** Top-view SEM images of the CsPbI_2_Br films with different cation-based IILs treatments. **e** PL and **f** TRPL spectra of CsPbI_2_Br perovskite films with different cation-based IILs treatments

The chemical structures of IILs with the same anions and different imidazolyl and alkyl groups are presented in Fig. S1. The IILs passivation layer was prepared by solution coating the IILs isopropanol solution onto perovskite film and annealing at 100 °C for 10 min. During the annealing process, the IILs could anchor to the CsPbI_2_Br through the coordination of N/F-atoms in the imidazolyl/anion and Pb in the perovskite [30]. Simultaneously, the hydrophobic alkyl chains of the IILs are arranged along the perovskite surface, which provides hydrophobicity to increase the moisture-resistance of the CsPbI_2_Br film [31]. In order to explore the effects of the IILs treatment on the crystallinity or orientation of the perovskite crystal structure, the XRD patterns of bare perovskite and perovskites modified by different IILs are characterized. As presented in Fig. 1a, only two main diffraction peaks from the (100) and (200) planes of the perovskite crystal structure can be observed, which is consistent with our previous reports [32, 33]. All the diffraction intensities of the (100) and (200) peaks were slightly enhanced in perovskite treated by IILs, while the peak positions have no obvious shift compared to bare perovskite film, indicating that the IILs could enhance the (100) orientation of perovskite but weren ‘t incorporated into the perovskite crystal lattice. According to the ultraviolet–visible (UV–vis) spectra of the perovskites (Fig. 1b), the absorption intensity and edge of the IILs-treated perovskite films have no discernible change, confirming that the crystal structure of the perovskite remains largely unchanged. The XRD and UV–vis results suggest that the IILs treatment does not change the cubic perovskite structure, and the IILs only remain at the surface and/or GBs of the perovskite film [34].

Next, time-of-flight secondary-ion mass spectrometry (ToF–SIMS) was performed under negative polarity to check the chemical distribution within the perovskite film with BMMIMBF_4_ treatment. As illustrated in Fig. 1c, it is found that the anions and cations of the IIL (BMMIMBF_4_) are not identically distributed in the perovskite film. Specifically, BMMIM^+^ is found to be distributed throughout the bulk film, while BF_4_^−^ is located mainly at the surface of the perovskite film. As shown in Fig. S2, the chemical distributions of N from BMMIM^+^ and F from BF_4_^−^ can be visualized and confirmed in the elemental 3D maps in the depth profile.

As shown in Fig. 1d, the surface morphologies of perovskite films with different IILs treatments were characterized with SEM measurements. After treatment with very dilute IILs solution, there was no significant change to the surface morphology of the perovskite film. However, the hydrophobic feature of the perovskite film was obviously improved, which is due to the hydrophobic alkyl chains and fluorinated anions. The hydrophobic feature can also be adjusted by the alkyl length and added side chains. The hydrophobic IIL layer deposited on the surface of the perovskite could prevent moisture and oxygen from infiltrating the perovskite layer, thereby improving device performance stability.

To obtain more information regarding the effect of IILs modification on the charge transfer process of CsPbI_2_Br film, steady-state PL measurements were conducted. As shown in Fig. 1e, all the PL intensities of the perovskite films with IILs modification are obviously quenched compared to that of the control film, mainly due to the quenching effect induced by charge transfer from the perovskite to the IILs [35]. In addition, when the spiro-OMeTAD layer was introduced, the PL quenching occurs more effectively in the IILs-treated perovskite films (Fig. S3). Further, time-resolved photoluminescence (TRPL) measurements were carried out to study the photogenerated carrier lifetime, as shown in Fig. 1f. Table S1 lists the fitting parameters of the TRPL curves fitted by a biexponential function. All the perovskite films with IILs treatment exhibit shorter average lifetimes than the bare perovskite film, which indicates the existence of accelerated charge transfer from the perovskite to the IILs [26]. As show in Fig. 1e, f and Table S1, the perovskite film with BMMIMBF_4_ treatment shows the most effective quenching efficiency and shortest PL decay time, indicating efficient charge transfer that would reduce the interfacial non-radiative recombination.

In order to clarify the possibility of charge transfer between the perovskite and IILs, UPS and electrochemical cyclic voltammetry (CV) characterization of perovskite and BMMIMBF_4_ IIL were conducted. As shown in Fig. S4, the Fermi level (*E*_F_) of the perovskite is close to the conduction band, indicating it is n-type, and the valence band maximum (VBM) is located at − 6.0 eV, while the highest occupied molecular orbital (HOMO) of BMMIMBF_4_ is at − 5.54 eV. When the n-type perovskite contacts the BMMIMBF_4_ under illumination, the holes will transfer from the perovskite to the BMMIMBF_4_, which results in the quenching of PL and decreased carrier lifetime. Therefore, IILs treatment leads to a faster charge transport and extraction at the perovskite surface, which means there is a possibility that charge-carrier recombination could be reduced [36] with potential benefit to the performance of the derived solar cells. To confirm this hypothesis, PSCs (FTO/TiO_2_/Perovskite/IILs/Au) without HTLs are fabricated (Fig. S5). Compared to the device without treatment, the IILs-treated device shows a remarkable increase in PCE from 5.16 to 9.86%, mainly driven by enhanced *V*_*oc*_ and fill factor (FF). This is indicative of reduced recombination and enhanced charge extraction at the perovskite/IILs interface, which is consistent with the PL and TRPL results (Fig. 1e, f) [12, 37–39].

**Fig. 2:**
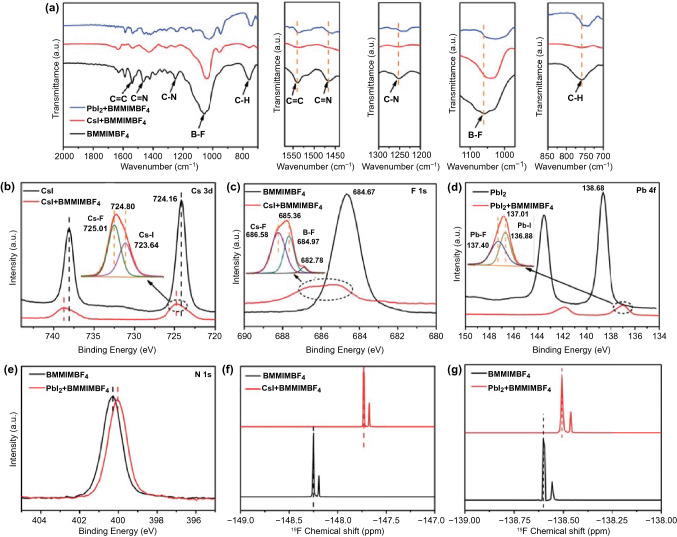
**a** FTIR spectra of BMMIMBF_4_ solution with or without PbI_2_/CsI additive. XPS spectra of **b** Cs 3d, **c** F 1 s of BMMIMBF_4_ solution with or without CsI additive. XPS spectra of **d** Pb 4f, and **e** N 1 s of BMMIMBF_4_ solution with or without PbI_2_ additive. **f** ^19^F NMR spectra of BMMIMBF_4_ solution with or without CsI additive. **g** ^19^F NMR spectra of BMMIMBF_4_ solution with or without PbI_2_ additive

In order to further investigate, the interaction between the IIL (BMMIMBF_4_) and CsPbI_2_Br perovskite, FTIR, XPS, and ^1^H, ^19^F, ^11^B NMR were conducted. Figure 2a shows the FTIR spectra of bare BMMIMBF_4_, BMMIMBF_4_ + PbI_2_, and BMMIMBF_4_ + CsI mixtures. The bond positions in the different mixtures are listed in Table S2. It is found that the stretching vibration peaks at 1252 and 1466 cm^−1^ assigned to the C–N and C=N bonds, respectively, in the pure BMMIMBF_4_ are shifted to lower wavenumbers of 1240 and 1457 cm^−1^ in BMMIMBF_4_ + PbI_2_. This indicates that the uncoordinated Pb^2+^ can form coordination bonds with C–N and C=N groups in BMMIMBF_4._ In contrast, the peaks for C–N and C=N bonds show almost no shift in BMMIMBF_4_ + CsI, indicating that there was no interaction between Cs^+^ and C–N/C=N bonds [40]. Meanwhile, the B-F peaks in the BMMIMBF_4_ + PbI_2_ and BMMIMBF_4_ + CsI mixtures show large red-shifts from 1056 cm^−1^ to 1025 and 1039 cm^−1^, respectively. This indicates that the uncoordinated Pb^2+^ and Cs^+^ can be effectively passivated by BF_4_^−^ through formation of ionic bonds. In addition, the bonding relationship between Pb^2+^(Cs^+^) and the IIL was also validated by the XPS spectra. As shown in Fig. 2b-e, when the IIL was mixed with CsI or PbI_2_, the binding energies of Cs 3d, Pb 4f, and N 1 s are all obviously shifted to lower position, while that of F 1 s is shifted to a higher position, which was attributed to the formation of strong bonding between the Cs^+^/Pb^2+^ and F atom in BF_4_^−^ and/or between Cs^+^ (Pb^2+^) and the electron-rich N atom in the alkyl chains. The formation of bonds increases the electron cloud density and decreases the electron affinity of Cs^+^ and Pb^2+^ ions. As shown in the inserts of Fig. 2b-d, all the Cs, F, and Pb peaks in the mixture split into two peaks, indicating that new ionic bonds (Cs-F and Pb-F) are formed between the CsI/PbI_2_ and BF_4_^−^ in the BMMIMBF_4._ These results imply that the strong bonding interaction between the BMMIMBF_4_ and perovskite is favorable and could provide passivation of uncoordinated Cs^+^/Pb^2+^ defects and the deep-level Pb-I antisite defects in perovskite through Cs-F and Pb-F bonds and suppress both the diffusion of inorganic cations and the phase transition of the inorganic perovskite crystal, thus significantly improving the stability of inorganic PSCs.

In order to gain deeper insights into the chemical interaction between the perovskite precursor and BMMIMBF_4_, a series of liquid state ^1^H, ^19^F, and ^11^B NMR spectra of BMMIMBF_4_ with PbI_2_ or CsI are presented in Figs. 2f, g, S6 and S7. With the addition of PbI_2_ or CsI into the BMMIMBF_4_ solution, as shown in Tables S3 and S4 in the Supporting Information, all the ^1^H peaks in the mixture had weak shifts compared with bare BMMIMBF_4_, suggesting that the coordinative bonds between N atoms in the alkyl chains and Pb (Cs) atoms in the PbI_2_ (CsI) are not so strong to affect adjacent CH_3_ or CH_2_ groups in the alkyl chains. In contrast, the characteristic F and B peaks of BF_4_^−^ show obvious shifts when mixed with PbI_2_ or CsI, due to the formation of Cs-F and Pb-F ionic bonds. From the aforementioned results, the interaction between BMMIMBF_4_ and inorganic perovskite CsPbI_2_Br most likely originates from the ionic bonds between BF_4_^−^ and Pb^2+^/Cs^−^ ions. The long alkyl chains and side methyl groups on the imidazole group lead to steric hindrance effects, hampering the interaction of the nitrogen atom on the imidazole with the PbI_2_ or CsI [41]. From examination of the results of NMR, FTIR, and XPS, it is concluded that the BMMIM^+^ cation in BMMIMBF_4_ could form a coordinative bond with uncoordinated Pb^2+^, while the anion (BF_4_^−^) could bond with Pb^2+^ and Cs^+^ through ionic bonding, which are favorable for inhibiting non-radiative recombination in CsPbI_2_Br perovskite.

**Fig. 3:**
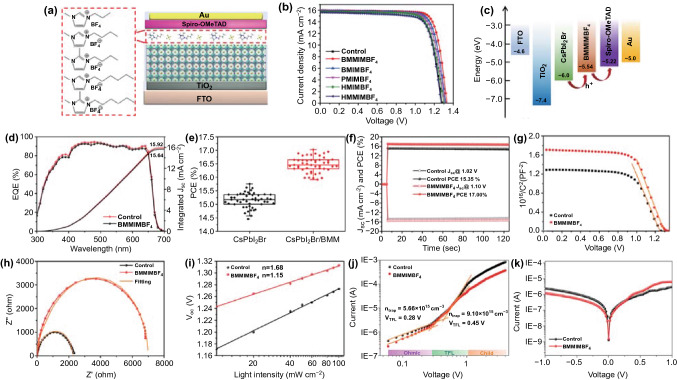
**a** Schematic image of a CsPbI_2_Br PSC with the structure FTO/TiO_2_/CsPbI_2_Br(IILs)/Spiro-OMeTAD/Au. **b** *J-V* curves and **c** schematic energy-level alignment of the CsPbI_2_Br PSC with BMMIMBF_4_ modification. **d** EQE spectra, **e** Box charts of PCE, **f** stable output curves, **g** Mott-Schottky plots, **h** Nyquist plots and **i** open-circuit voltage dependence on light intensity of the CsPbI_2_Br PSC with BMMIMBF_4_ modification. **j** Space-charge-limited current versus voltage for the FTO/TiO_2_/CsPbI_2_Br/PCBM/Ag and FTO/TiO_2_/CsPbI_2_Br(BMMIMBF_4_)/PCBM/Ag devices. **k** *J-V* curves under dark conditions of the CsPbI_2_Br PSC without or with BMMIMBF_4_ modification

To comprehensively evaluate the effect of the IILs treatment on the photovoltaic performance of CsPbI_2_Br PSCs, as shown in Fig. 3a, an n-i-p device with the planar structure glass/FTO/TiO_2_/CsPbI_2_Br(IILs)/Spiro-OMeTAD/Au was fabricated, where the IILs with different cations were coated onto the perovskite layers as surface passivators. Figure 3b and Table 1 present the current density–voltage (*J-V*) curves and the corresponding photovoltaic parameters of optimized devices. The device with BMMIMBF_4_ treatment delivers a champion PCE as high as 17.02%, with short-circuit current density (*J*_sc_) of 15.96 mA cm^−2^, *V*_oc_ of 1.33 V, and FF of 80.08%. Meanwhile, the best control device shows an inferior PCE of 15.62%, with *J*_sc_ of 15.81 mA cm^−2^, *V*_oc_ of 1.28 V, and FF of 77.10%. As shown in Fig. S8 and Tables S5 and S6, the dependence of the device performance on the concentration of the BMMIMBF_4_ treatment was also investigated. The optimized concentration of BMMIMBF_4_ is 0.03 wt%. The schematic energy-band alignment of the CsPbI_2_Br PSCs with BMMIMBF_4_ treatment is illustrated in Fig. 3c, where the VBM of perovskite is located at − 6.0 eV, and the HOMO of BMMIMBF_4_ is at − 5.54 eV, which leads to a better energy-level alignment with the HOMO (− 5.22 eV) of Spiro-OMeTAD. Such a band alignment between the CsPbI_2_Br surface and Spiro-OMeTAD facilities hole transport and reduces interfacial recombination [3, 33]. EQE spectra of devices are shown in Fig. 3d. The integrated current density values for control and BMMIMBF_4_-treated devices are 15.64 and 15.92 mA cm^−2^, respectively, which coincide with the *J*_sc_ values derived from the *J-V* measurements. Figures 3e and S9 exhibit the statistical photovoltaic parameter distributions of 50 individual devices with or without BMMIMBF_4_ treatment, which permit the conclusion that BMMIMBF_4_ treatment can undoubtedly boost the performance of PSCs, mainly stemming from improved *V*_oc_ and FF. The stabilized PCEs were further measured with devices biased at the initial maximum power point voltage for 120 s. As shown in Fig. 3f, the BMMIMBF_4_-treated device achieved the stabilized PCE of 17.00% at the maximum power point (1.10 V), which is in good agreement with the PCE obtained from the *J-V* measurements.

**Table 1:** Summary of the photovoltaic parameters of the CsPbI_2_Br PSCs treated using IILs with different cations

Sample	*V*_oc_ (V)	*J*_sc_ (mA cm^−2^)	FF (%)	PCE (%)
Control	1.28	15.81	77.10	15.62
BMMIMBF_4_	1.33	15.96	80.08	17.02
BMIMBF_4_	1.32	15.93	78.21	16.45
PMIMBF_4_	1.31	15.78	77.74	16.09
HMIMBF_4_	1.30	15.77	76.33	15.66
HMMIMBF_4_	1.27	15.62	76.74	15.25

A Mott-Schottky analysis was conducted to study the built-in potential (*V*_bi_) in the devices. As illustrated in Fig. 3g, the device with BMMIMBF_4_ treatment shows a *V*_bi_ of 1.29 V, which is larger than that of the control device (1.25 V). The larger *V*_bi_ in the treated device is usually related to a higher *V*_oc_ value. Electrochemical impedance spectroscopy (EIS) was conducted under dark conditions (Fig. 3h) [33, 42]. The fitted parameters are summarized in Table S7. Compared with the control device, the charge transport resistance (*R*_ct_) of the device with BMMIMBF_4_ treatment was decreased significantly from 754.7 to 493.9 Ω, while the recombination resistance (*R*_rec_) was increased significantly from 2.35 to 6.89 kΩ. The smaller *R*_ct_ and larger *R*_rec_ suggest remarkably enhanced carrier transfer and suppressed charge recombination due to low defect density in the modified device, resulting in the enhanced *V*_oc_ and FF [43, 44].

To further understand the mechanism of trap-assisted carrier recombination during device operation, the relationship between *V*_oc_ and light intensity was measured (Fig. 3i), which yielded ideality factors (IFs) of the control and treated devices of 1.68 and 1.15 K_B_T/q, respectively. The largely reduced IF indicates that IILs treatment can effectively reduce the trap-assisted non-radiative recombination, which is favorable for obtaining high *V*_oc_ and FF values [45–47].

To quantitatively evaluate the affects of IILs modification on the trap density of perovskite films, a space-charge limited current (SCLC) characterization was conducted on an electron-transport device (ITO/TiO_2_/perovskite/PCBM/Ag) [48, 49]. As shown in Fig. 3j, the trap-filled limit voltage (V_TFL_) of the device with IILs treatment is ~ 0.28 V, corresponding to a relative trap density (*n*_*t*_) of 5.66 × 10^15^ cm^−3^, while the control device displays a higher V_TFL_ of 0.45 V with *n*_*t*_ of 9.1 × 10^15^ cm^−3^. The lower defect density induced by the IILs treatment could be attributed to the effective passivation of the perovskite film by the IILs. Therefore, the SCLC results are consistent with the previous EIS and *V*_oc_ versus light analysis, in which the perovskite film treated with IILs shows a lower defect density and offers an enhanced V_oc_ and FF in the PSCs. To evaluate the charge transport properties of the devices, dark *J-V* curves were measured and are shown in Fig. 3k. The device with IIL treatment has a lower leakage current than the control one, indicating a decreased carrier generation rate and reduced background carrier density in the device. Because the carrier generation rate in a solar cell in the dark is related to the trap density in the device [3, 50, 51], the dark *J-V* result further indicates that the IIL treatment could effectively passivate defects in perovskite.

**Fig. 4:**
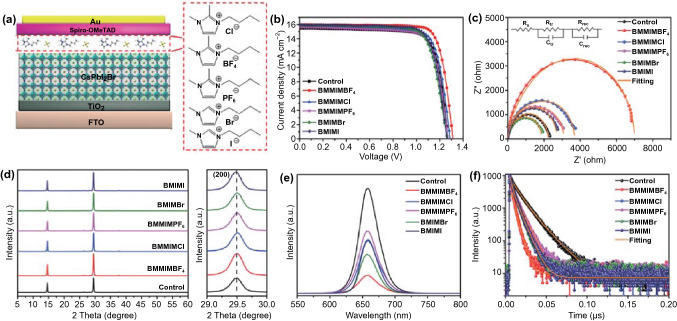
**a** Schematic image of a CsPbI_2_Br PSC with the structure FTO/TiO_2_/CsPbI_2_Br(IILs)/Spiro-OMeTAD/Au. **b** *J-V* curves and **c** Nyquist plots of CsPbI_2_Br treated using IILs with different anions. **d** XRD patterns and (200) diffraction peaks, **e** PL and **f** TRPL spectra of CsPbI_2_Br perovskite films treated using IILs with different anions

The influence of IILs with different anions on the perovskite devices and films was also studied. As shown in Fig. 4a, IILs with different anions were coated onto the perovskite layer as surface passivators. Figure 4b and Table 2 present the *J-V* curves and the corresponding photovoltaic parameters of optimized CsPbI_2_Br PSCs. The device with BMMIMBF_4_ treatment delivers a champion PCE as high as 16.89%. The EIS curves (Fig. 4c) and their fitted parameters (Table S8) suggest remarkably enhanced carrier transfer and suppressed charge recombination in the BMMIMBF_4_-treated devices. The XRD patterns and UV–vis spectra of perovskite films with IILs treatment are shown in Fig. 4d and S10, respectively. All the diffraction peaks and absorption edges of the perovskite films have no discernible change, confirming that the crystal structure of the perovskite remains largely unchanged by IILs modification. The PL and TRPL spectra of perovskite films with different IILs treatments were measured and are shown in Fig. 4e, f, respectively. All the PL spectra of these perovskite films show significant quenching and shorter carrier lifetime compared to those of the bare perovskite film, indicating increased charger transfer from the perovskite to the IILs. Among all these perovskite films, the one with BMMIMBF_4_ treatment shows the strongest quenching and the shortest carrier lifetime (Table S9), indicating the most effective charger transfer process.

**Table 2:** Summary of the photovoltaic parameters of the CsPbI_2_Br PSCs treated using IILs with different anions

Sample	*V*_oc_ (V)	*J*_sc_ (mA cm^−2^)	FF (%)	PCE (%)
Control	1.27	15.45	77.86	15.28
BMMIMBF_4_	1.31	15.95	80.63	16.89
BMMIMCl	1.29	15.77	77.57	15.78
BMMIMPF_6_	1.27	15.62	76.74	15.25
BMIMBr	1.26	15.88	76.27	15.32
BMIMI	1.26	15.97	77.85	15.67

**Fig. 5:**
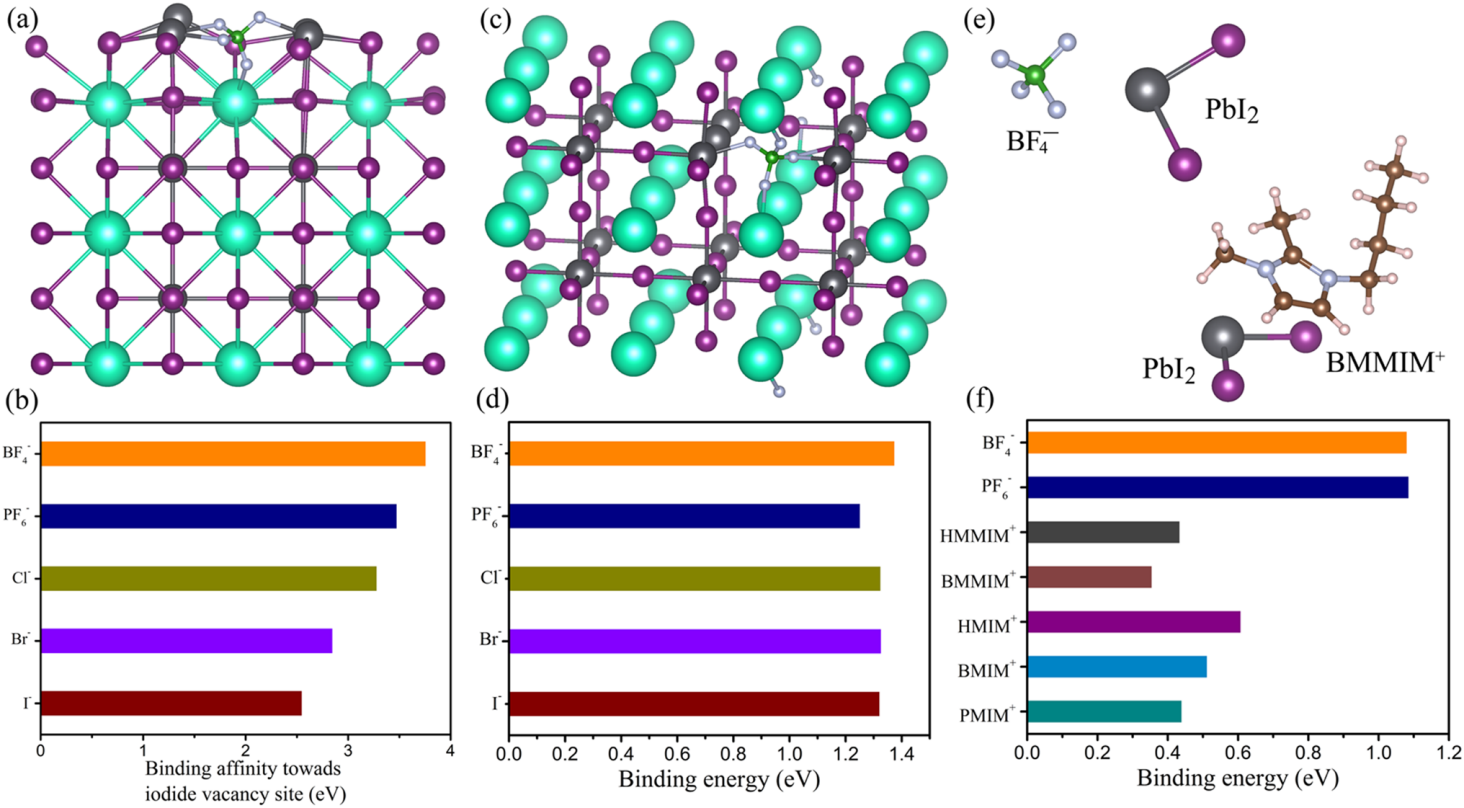
**a** Calculated structure illustrating the passivation of an I^−^ vacancy at the CsPbI_2_Br surface by a BF_4_^−^ anion. **b** The relative interaction strengths of different anions with the I^−^ vacancy at the surface of the perovskite. **c** Calculated structure illustrating the interaction of Pb-I antisite with BF_4_^−^. **d** The binding energy of different anions with Pb-I antisite defect. **e** Calculated structure illustrating the interaction of PbI_2_ with BF_4_^−^ or BMMIM^+^. **f** The binding energy of different ions with PbI_2_

Upon IILs treatment, as demonstrated by the FTIR, XPS, and NMR results, the IILs could form strong ionic bonds with the uncoordinated Pb and Cs to passivate positively charged halide vacancies [52, 53]. As such, the I^−^ vacancies were diminished, accompanied by reduced defect density. In order to further demonstrate the passivation effect of the IILs, DFT calculations were carried out to adequately clarify the interaction the anions in the IILs with halide vacancies [54–57]. For reducing the amount of calculation, a 2 × 2 × 3 supercell of CsPbI_3_ was employed in the study (The detailed models and calculation details are given in the experimental section). Figure 5a shows a calculated structure that illustrates a BF_4_^−^ anion passivating an I^−^ vacancy at the CsPbI_2_Br surface. I^−^ vacancy defects often act as non-radiative recombination centers and are responsible for the ionic conductivity of perovskites, causing operational instability. The relative binding affinities of different anions in the IILs to I^−^ vacancies at the surface were estimated. The results exhibited in Fig. 5b reveal that BF_4_^−^ has the highest binding energy to vacant I^−^ sites in comparison with PF_6_^−^, Cl^−^, Br^−^, and I^−^. Pb-I antisites form localized states near the valence band edge of perovskite and act as non-radiative recombination centers. The binding energy between different anions in the IILs and Pb-I antisite was also confirmed by DFT. Figure 5c shows a calculated structure that illustrates a BF_4_^−^ anion passivating an Pb-I antisite in the CsPbI_2_Br perovskite. As shown in Fig. 5d, BF_4_^−^ has the highest binding energy to Pb-I antisite in comparison with other anions, which means that Pb-I antisite could be more effectively passivated by BF_4_^−^. Furthermore, the bonding energies of anions and cations in IILs with PbI_2_ are also calculated to identify the binding preference of the ions in IILs with CsPbI_2_Br. Figure 5e shows the calculated structures for PbI_2_ binding with a BF_4_^−^ anion or BMMIM^+^ cation. As shown in Fig. 5f, the binding energy is as high as 0.61 eV for the HMIM^+^ cation, which is the highest among the cations in this study. However, it is still much smaller than that of the anions BF_4_^−^ (1.08 eV) and PF_6_^−^ (1.09 eV). The calculation result shows that the PMIM^+^, BMIM^+^, HMIM^+^, BMMIM^+^, and HMMIM^+^ cations cannot interact directly with PbI_2_, while BF_4_^−^ or PF_6_^−^ can strongly interact with PbI_2_. This result explicitly shows that the BF_4_^−^ or PF_6_^−^ anions can be incorporated into the CsPbI_3_ perovskite at the film surface to eliminate I^−^ vacancies to reduce interfacial non-radiative recombination. In addition, compared with BF_4_^−^ (2.76 Å), the larger size of PF_6_^−^ (3.00 Å) could induce a certain stress to weaken the interaction energy with the perovskite film [58–60]. Therefore, BF_4_^−^ anion is more suitable for passivating CsPbI_2_Br perovskite films. From the combined XPS, FTIR, NMR, and DFT calculated results, it can be concluded that anions (BF_4_^−^) play a more important role in passivating uncoordinated Pb^+^, Pb-I antisite defects and eliminating I^−^ vacancy defects than do the cations.

**Fig. 6:**
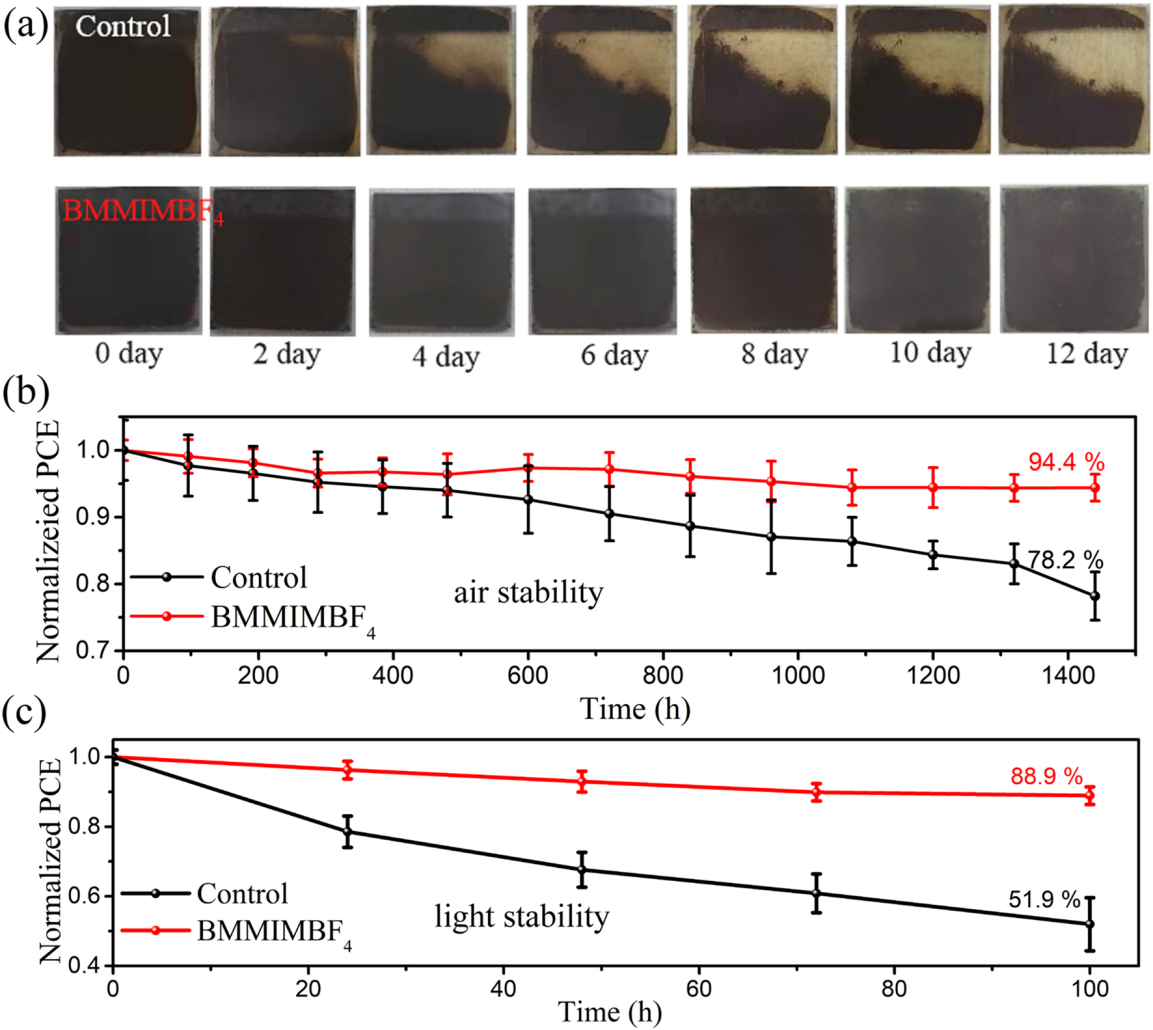
**a** Photographs of control and BMMIMBF_4_-treated CsPbI_2_Br films aged in ambient air conditions (RH: ~ 25%, T = 25 °C). **b** Air stability and **c** light stability of the CsPbI_2_Br PSCs with or without BMMIMBF_4_ treatment

Apart from the efficiency, the device stability was evaluated upon exposure to air and light conditions. The appearance of the perovskite films exposed in air for different times was tracked, as seen in the photographs in Fig. 6a. The BMMIMBF_4_-treated film remained black even after 12 days exposure in air, while the control film shows obvious fading near the edges, indicating a phase transition from α to δ. As expected, the CsPbI_2_Br solar cells with BMMIMBF_4_ treatment show remarkably superior stability compared to the control one, retaining 94.4% of their initial efficiencies after being stored in air for 1440 h under ~ 25% RH. In contrast, the control device lost more than 20% of its PCE over the same period (Fig. 6b). Most importantly, as shown in Fig. 6c, the photostability of the devices with IILs treatment was significantly improved, with ~ 90% of their initial efficiencies remaining after 100 h under AM 1.5G sun illumination at 40 °C in ambient air (RH: ~ 30%), while the PCE of the control device decreased by nearly 50%. Therefore, the IILs surface passivation could significantly improve the ambient and light stability of the CsPbI_2_Br PSCs, which can be ascribed to the following effects: (1) The long alkyl chain of the IIL enhances the hydrophobicity of the perovskite film as confirmed by the dramatically increased contact angle from 64.9° to 91.2° (Fig. 1d) [25]. (2) Effective defect passivation reduces the active sites for phase transition.

## Conclusions

A series of IILs with different cations and anions has been applied to unravel the mechanism for passivation of CsPbI_2_Br inorganic perovskites by the IILs. In contrast to previous studies, this work found that anions of the IILs play a more important role in passivation of lead- and cesium-related defects in inorganic perovskite compared with imidazole cations because they can form strong ionic interactions (Pb-F, Cs-F). These ionic bonds could passivate the surface and grain boundaries of the perovskite to reduce the charge-carrier recombination at the CsPbI_2_Br/spiro-OMeTAD interface. Due to the large steric hindrance effect, the interaction between the large imidazole cations and lead defects is weakened. Meanwhile, the IILs could improve the energy-level alignment between the perovskite and Spiro-OMeTAD for efficient charge transfer. Further, the IILs modification improves the hydrophobicity of the perovskite film, leading to an improved air stability of PSCs. As a result, the optimized CsPbI_2_Br PSCs with BMMIMBF_4_ treatment achieve the highest PCE of 17.02%, which is much higher than the PCE of the control device (15.62%). The unencapsulated device modified with BMMIMBF_4_ presents outstanding long-term stability when stored in ambient air at 25 °C with a RH of 25%, or under continuous illumination for 100 h. This work provides insightful guidelines toward the design or choice of effective IILs for improving the performance of inorganic PSCs or related photoelectric devices.

## Supplementary Information

Below is the link to the electronic supplementary material.

Supplementary file1 (PDF 1192 KB)
