# Supplementary material for: Unraveling Passivation Mechanism of Imidazolium-Based Ionic Liquids on Inorganic Perovskite to Achieve Near-Record-Efficiency CsPbI2Br Solar Cells
Source: Nanomicro Lett. 2021 Dec 2;14:7. doi: 10.1007/s40820-021-00763-8 (PMC8639893; doi:10.1007/s40820-021-00763-8)
Supplement: Supplementary file 1 — Supplementary file1 (PDF 1192 KB) [file 40820_2021_763_MOESM1_ESM.pdf]

Supporting Information for

Unraveling Passivation Mechanism of Imidazolium-Based Ionic Liquids on Inorganic Perovskite to Achieve Near-Record-Efficiency $CsPbI_2Br$ Solar Cells

Jie Xu[1], Jian Cui[1], Shaomin Yang[1], Yu Han[1], Xi Guo[1], Yuhang Che[1], Dongfang Xu[1], Chenyang Duan[1], Wenjing Zhao[1], Kunpeng Guo[3], Wanli Ma[4], Baomin Xu[5], Jianxi Yao[6], Zhike Liu[1,*], Shengzhong (Frank) Liu[1,2,*]

[1]Key Laboratory of Applied Surface and Colloid Chemistry, Ministry of Education; Shaanxi Key Laboratory for Advanced Energy Devices; Shaanxi Engineering Lab for Advanced Energy Technology, School of Materials Science and Engineering, Shaanxi Normal University, Xi'an, 710119, P. R. China

[2]iChEM, Dalian Institute of Chemical Physics, University of Chinese Academy of Sciences, Dalian National Laboratory for Clean Energy, Chinese Academy of Sciences, Dalian, 116023, P. R. China

[3]Ministry of Education Key Laboratory of Interface Science and Engineering in Advanced Materials, Research Center of Advanced Materials Science and Technology, Taiyuan University of Technology, Taiyuan, 030024, P. R. China

[4]Institute of Functional Nano & Soft Materials, Jiangsu Key Laboratory for Carbon-Based Functional Materials & Devices, Joint International Research Laboratory of Carbon-Based Functional Materials and Devices, Soochow University, Suzhou 215123, P. R. China

[5]Department of Materials Science and Engineering and Shenzhen Engineering Research and Development Center for Flexible Solar Cells, Southern University of Science and Technology, Shenzhen 518055, P. R. China

[6]State Key Laboratory of Alternate Electrical Power System with Renewable Energy Sources North China Electric Power University, Beijing, 102206, P. R. China

*Corresponding authors. E-mail: zhike2015@snnu.edu.cn (Zhike Liu); szliu@dicp.ac.cn (Shengzhong (Frank) Liu)

Supplementary Figures and Tables

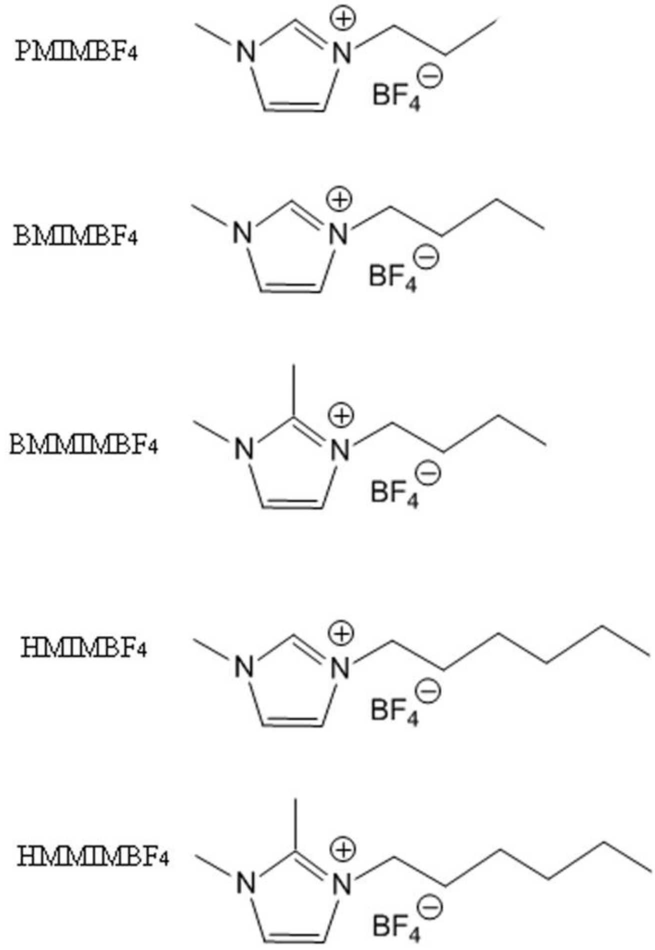

Fig. S1 Chemical formula of different imidazole-based ionic liquid

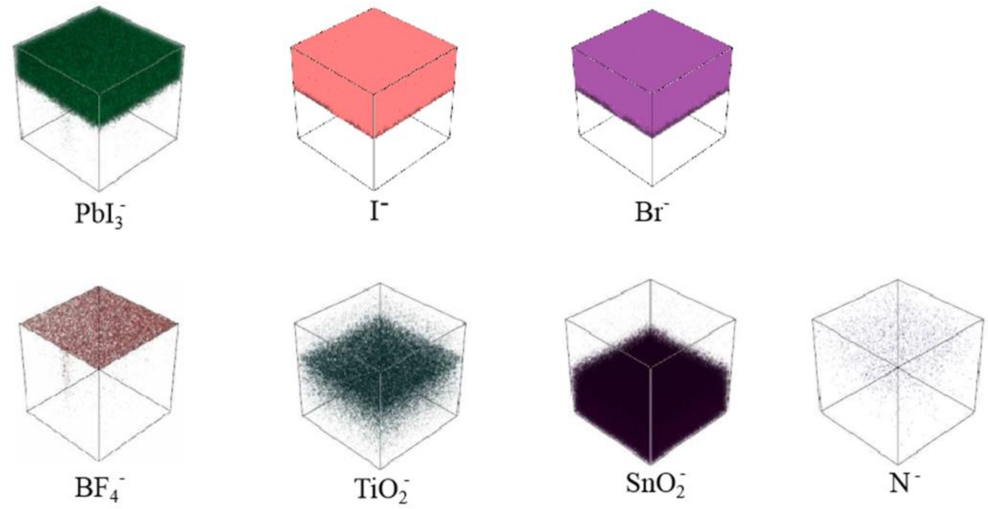

Fig. S2 3D reconstructed images of PbI_3^-, I^-, Br^-, BF_4^-, TiO_2^-, SnO_2^- and N^- in $CsPbI_2Br$/$BMMIMBF_4$ perovskite film as obtained from the TOF-SIMS analysis

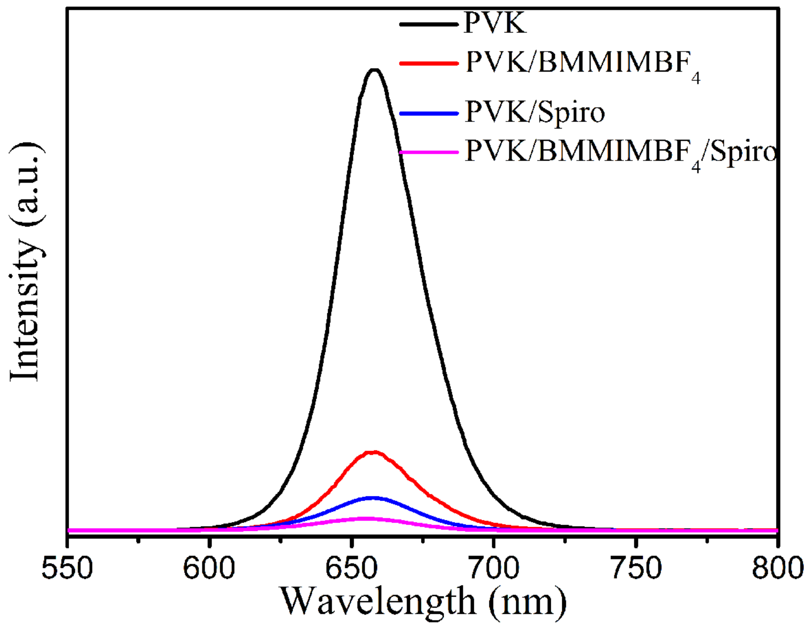

Fig. S3 PL spectra of $CsPbI_2Br$ film, $CsPbI_2Br/BMMIMBF_4$ film, $CsPbI_2Br$-Spiro film and $CsPbI_2Br/BMMIMBF_4$/Spiro film

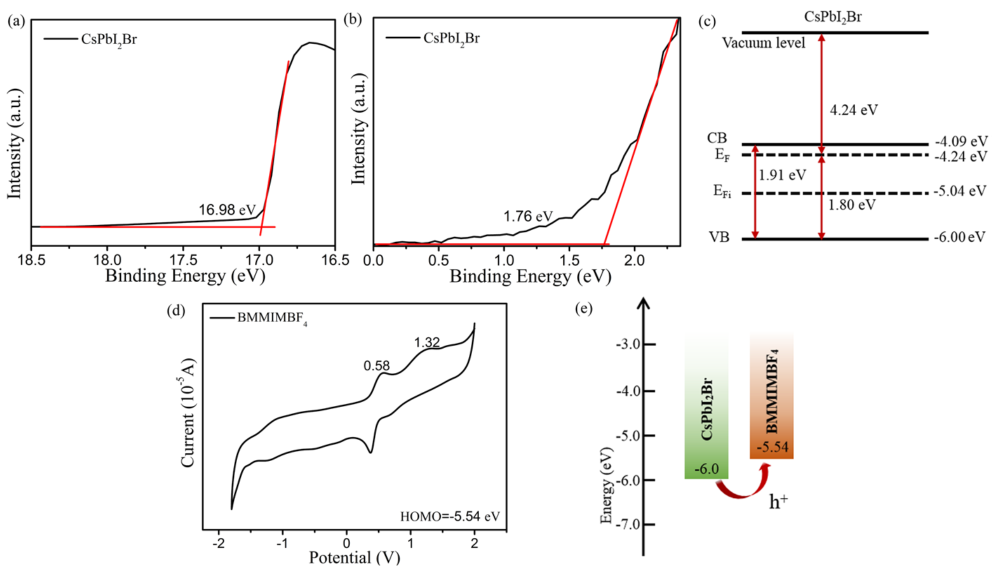

Fig. S4 UPS spectra of (**a, b**) $CsPbI_2Br$ film. **c**) Energy level diagrams for the $CsPbI_2Br$ film. **d**) Cyclic voltammograms spectra for the $BMMIMBF_4$. (**e**) Schematic energy level alignment of $CsPbI_2Br$ film and $BMMIMBF_4$

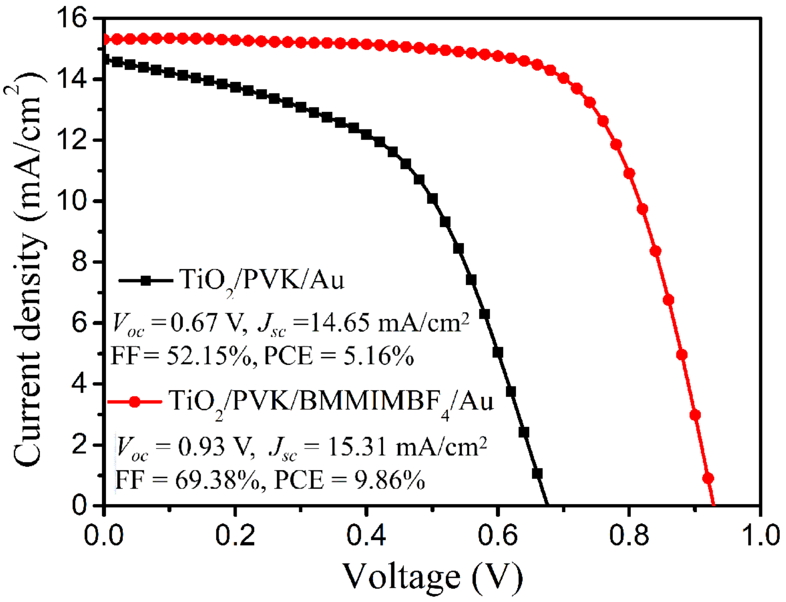

Fig. S5 *J*-*V* curves of the FTO/TiO_2/Perovskite/Au and FTO/TiO_2/Perovskite($BMMIMBF_4$)/Au devices

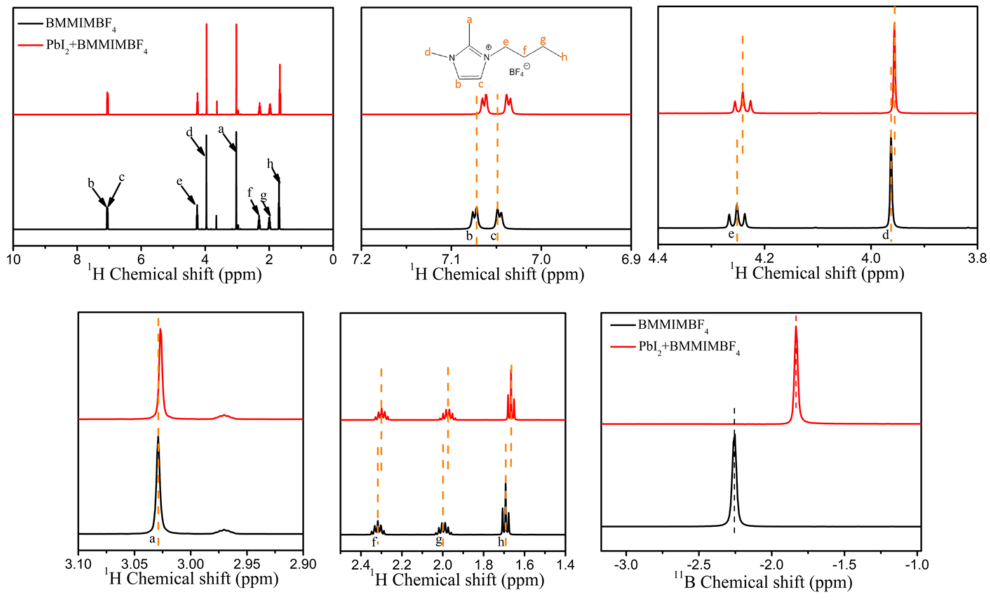

Fig. S6 ^{1}H NMR and ^{11}B NMR of $BMMIMBF_4$ solution with or without PbI_2 additive

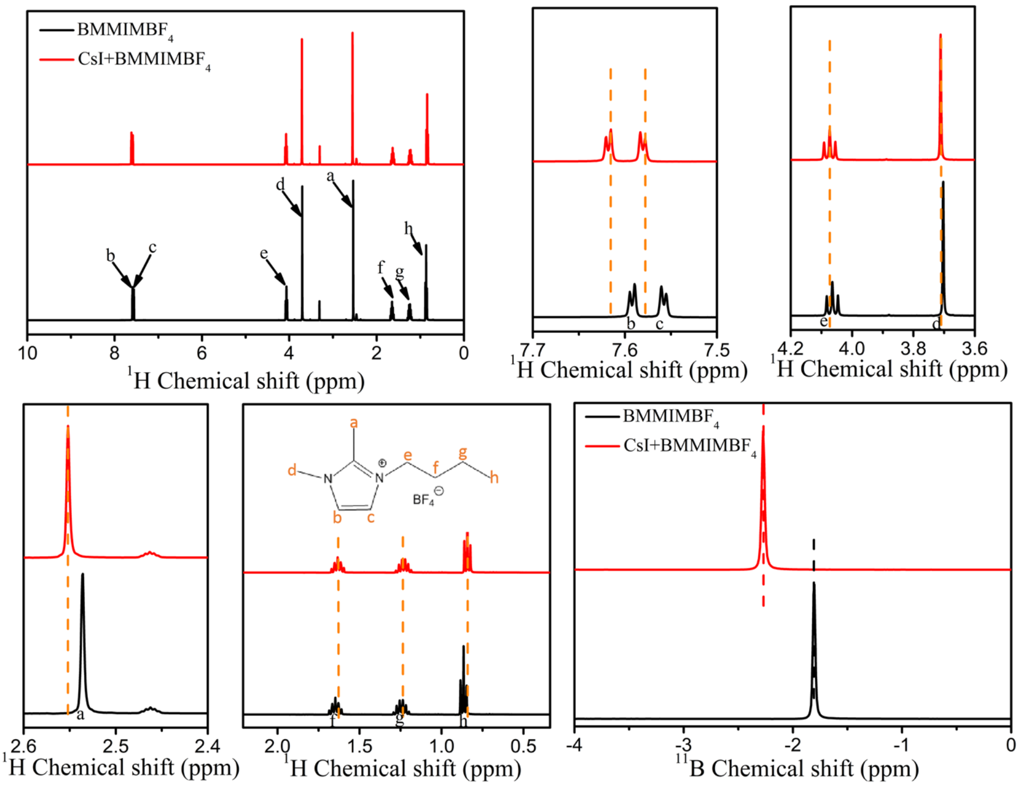

Fig. S7 ^{1}H NMR and ^{11}B NMR of $BMMIMBF_4$ solution with or without CsI additive

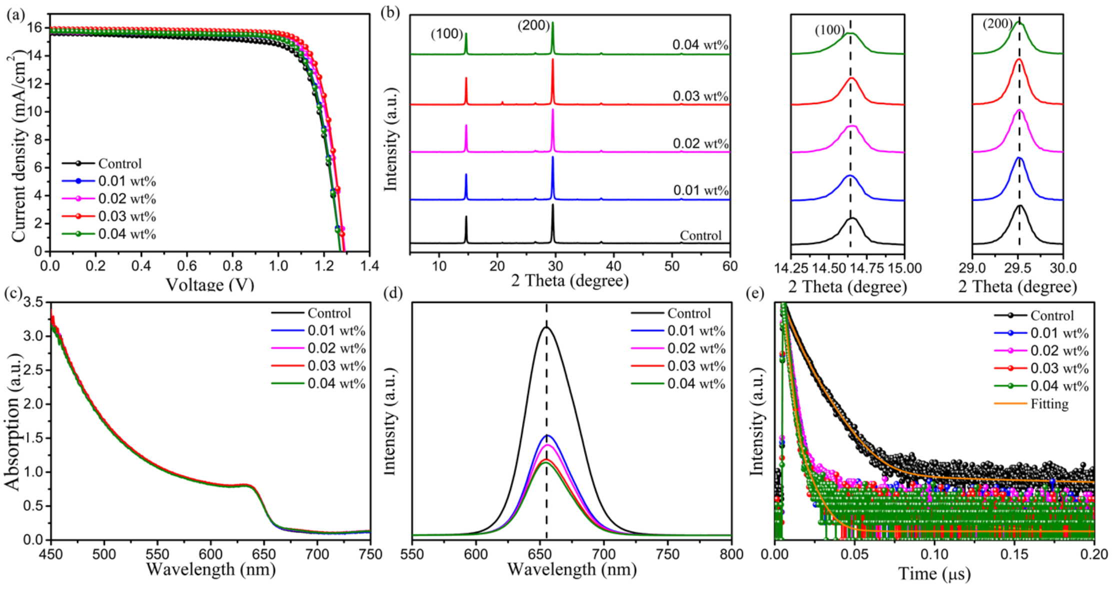

Fig. S8 (**a**) *J-V* curves of the $CsPbI_2Br$ PSCs with different concentrations of IILs treatment. (**b**) XRD patterns, (**c**) UV-vis absorption spectra, (**d**) PL and (**e**) TRPL spectra of $CsPbI_2Br$ perovskite films with different concentrations of $BMMIMBF_4$ treatment

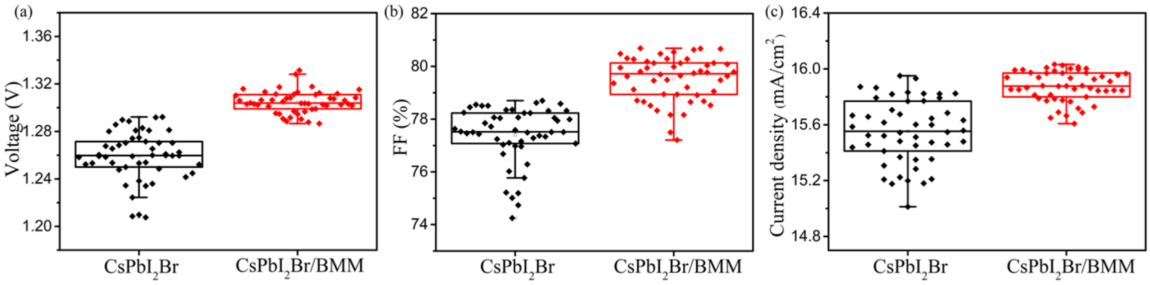

Fig. S9 Box charts of (**a**) V_{oc}, (**b**) FF, (**c**) J_{sc} of the PSCs (50 samples) with $BMMIMBF_4$ treatment

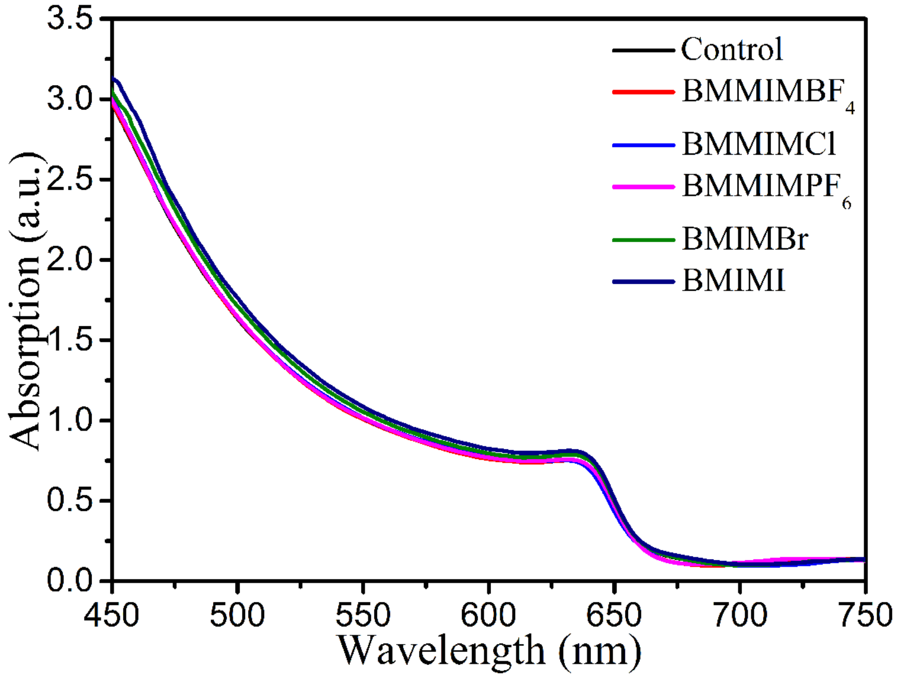

Fig. S10 UV-vis absorption spectra of $CsPbI_2Br$ film with different anion-based IILs treatment

Table S1 Fitting parameters of the TRPL spectra for the $CsPbI_2Br$ films with different cation-based IILs treatment

Samples	A_1 (%)	τ_1 (ns)	A_2 (%)	τ_2 (ns)	τ_{ave} (ns)
Control	35.76	16.54	64.24	7.87	12.54
$BMIMBF_4$	63.58	1.81	36.42	4.75	3.58
$BMMIMBF_4$	28.52	5.05	71.48	2.16	3.56
$PMIMBF_4$	49.57	7.06	50.43	2.64	5.84
$HMIMBF_4$	56.78	3.60	43.22	7.69	6.13
$HMMIMBF_4$	48.54	3.79	51.46	10.37	8.68

Table S2 FTIR wavenumber positions of different chemical bonds of $BMMIMBF_4$ with or without PbI_2 or CsI additive

Samples	C=C (cm^{-1})	C-N (cm^{-1})	C=N (cm^{-1})	B-F (cm^{-1})	C-H (cm^{-1})
$BMMIMBF_4$	1539	1252	1466	1056	757
PbI_2+$BMMIMBF_4$	1536	1240	1457	1025	747
CsI+$BMMIMBF_4$	1538	1251	1467	1039	757

Table S3 NMR Relative chemical shift of $BMMIMBF_4$ with or without PbI_2 additive

Samples	H_b (ppm)	H_c (ppm)	H_e (ppm)	H_d (ppm)	H_a (ppm)	H_f (ppm)	H_g (ppm)	H_h (ppm)	F (ppm)	B (ppm)
$BMMIMBF_4$	7.07	7.04	4.25	3.96	3.03	2.31	1.99	1.69	-138.6	-2.25
PbI_2+$BMMIMBF_4$	7.06	7.03	4.24	3.95	3.02	2.29	1.97	1.66	-138.5	-1.83
Relative chemical shift	0.01	0.01	0.01	0.01	0.01	0.02	0.02	0.03	0.1	0.42

Table S4 NMR Relative chemical shift of $BMMIMBF_4$ with or without CsI additive

Samples	Hb (ppm)	Hc (ppm)	He (ppm)	Hd (ppm)	Ha (ppm)	Hf (ppm)	Hg (ppm)	Hh (ppm)	F (ppm)	B (ppm)
$BMMIMBF_4$	7.62	7.56	4.06	3.70	2.53	1.63	1.23	0.84	-148.25	-1.79
CsI+$BMMIMBF_4$	7.59	7.58	4.07	3.71	2.55	1.64	1.24	0.85	-147.73	-2.29
Relative chemical shift	0.03	0.02	0.01	0.01	0.02	0.01	0.01	0.01	0.42	0.50

Table S5 Summary of the photovoltaic parameters of the $CsPbI_2Br$ PSCs with different concentrations of $BMMIMBF_4$ treatment

$BMMIMBF_4$	V_{oc} (V)	J_{sc} (mA/cm^2)	FF (%)	PCE (%)
Control	1.27	15.62	76.74	15.25
0.01 wt%	1.27	15.75	78.72	15.77
0.02 wt%	1.29	15.74	78.78	16.01
0.03 wt%	1.29	15.91	80.48	16.47
0.04 wt%	1.27	15.77	78.53	15.73

Table S6 Fitting parameters of the TRPL spectra for the $CsPbI_2Br$ films with different concentrations of $BMMIMBF_4$ treatment

Samples	A_1 (%)	τ_1 (ns)	A_2 (%)	τ_2 (ns)	τ_{ave} (ns)
Control	61.41	22.76	38.59	7.14	20.19
0.01 wt%	87.70	4.46	12.30	11.7	6.42
0.02 wt%	30.39	8.92	69.61	3.52	6.36
0.03 wt%	11.57	9.35	88.43	3.57	5.04
0.04 wt%	88.85	2.52	11.15	7.22	3.76

Table S7 EIS fitting parameters for the $CsPbI_2Br$ and $CsPbI_2Br/BMMIMBF_4$ PSCs

Samples	R_s (Ω)	R_{tr} (Ω)	C_{tr} (F)	R_{rec} (kΩ)	C_{rec} (F)
Control	2.87	754.7	1.10×10^{-7}	2.35	1.10×10^{-7}
$BMMIMBF_4$	2.43	493.9	1.46×10^{-7}	6.89	7.44×10^{-7}

Table S8 EIS fitting parameters for the $CsPbI_2Br$ PSCs with different anion-based IILs treatment

Samples	R_s (Ω)	R_{tr} (Ω)	C_{tr} (F)	R_{rec} (kΩ)	C_{rec} (F)
Control	2.87	754.7	1.10×10^{-7}	2.35	1.10×10^{-7}
$BMMIMBF_4$	2.43	493.9	1.46×10^{-7}	6.89	7.44×10^{-7}
BMMIMCl	3.03	715.4	1.28×10^{-7}	3.68	2.79×10^{-7}
$BMMIMPF_6$	2.12	852.2	1.06×10^{-7}	3.06	2.11×10^{-7}
BMIMBr	5.29	903.4	1.04×10^{-7}	2.62	1.76×10^{-7}
BMIMI	3.89	887.6	1.05×10^{-7}	2.89	2.76×10^{-7}

Table S9 Fitting parameters of the TRPL spectra for the $CsPbI_2Br$ films with different anion-based IILs treatment

Samples	A_1 (%)	τ_1 (ns)	A_2 (%)	τ_2 (ns)	τ_{ave} (ns)
Control	35.76	16.54	64.24	7.87	12.54
$BMMIMBF_4$	47.00	6.58	53.00	2.38	5.36
BMMIMCl	48.24	7.41	51.76	3.14	6.07
$BMMIMPF_6$	51.36	8.30	48.64	2.68	6.98
BMIMBr	44.62	7.00	55.38	3.19	5.6
BMIMI	49.06	7.20	50.94	2.64	5.9
